# A modified intrascleral intraocular lens fixation technique with fewer anterior segment manipulations: 27-gauge needle-guided procedure with built-in 8–0 absorbable sutures

**DOI:** 10.1186/s12886-019-1239-2

**Published:** 2019-11-21

**Authors:** Yuan Yang, Teng-teng Yao, Ya-li Zhou, Yi-xiao Wang, Zhao-yang Wang

**Affiliations:** 1 Department of Ophthalmology, Shanghai Ninth People’s Hospital, Shanghai Jiao Tong University School of Medicine, No. 639 Zhizaoju Road, Shanghai, 200011 China; 2 Shanghai Key Laboratory of Orbital Diseases and Ocular Oncology, No. 639 Zhizaoju Road, Shanghai, 200011 China

**Keywords:** Intrascleral intraocular lens fixation, Flange, 8–0 absorbable suture, 27-gauge round needle

## Abstract

**Background:**

To report a modified surgical technique for intrascleral intraocular lens (IOL) fixation with fewer anterior segment manipulations in eyes lacking sufficient capsular support.

**Methods:**

Eyes from 14 patients who underwent 27-gauge needle-guided intrascleral IOL fixation with built-in 8–0 absorbable sutures were studied. The 8–0 absorbable sutures were inserted into 27-gauge round needles and used to create sclerotomies at the 4 o’clock and 10 o’clock positions under the scleral flap. The sutures were used to tie knots at the end of each haptic and guide haptic externalization through the sclerotomy. After externalization, a sufficient flange was created at the end of each haptic and fixed under the scleral flaps. The best corrected visual acuity (BCVA), corneal endothelial cell density (ECD), IOL tilt and decentration, previous surgery history, and complications were determined.

**Results:**

Fourteen cases were analyzed. The majority of eyes exhibited an improvement in the BCVA after surgery. When comparing the last follow-up to preoperative visual acuity, the mean change in BCVA was + 26.32 letters (*p* = 0.011). Postoperative complications included postoperative hypotony in 3 eyes, ocular hypertension in 2 eyes. No cases of postoperative cystoid macular edema (CME), vitreous hemorrhage (VH), IOL dislocation, or endophthalmitis were observed.

**Conclusions:**

The 27-gauge needle-guided intrascleral IOL fixation technique with built-in 8–0 absorbable sutures is easy to perform with fewer anterior chamber manipulations and achieves both anatomical and optical stability.

## Background

Surgical techniques for intraocular lens (IOL) implantation in an eye without sufficient posterior capsular support include anterior chamber IOL (ACIOL) implantation, iris-fixed IOL implantation, or intrascleral-fixated IOL implantation [1–5]. ACIOL or iris-fixed IOL implantation is convenient but might lead to corneal decompensation, iris chafing, uveitis-glaucoma-hyphema (UGH) syndrome or cystoid macular edema (CME) [6, 7]. Intrascleral-fixated IOL implantation therefore has some relative advantage with its more physiological location. It reduces the risk of corneal decompensation, peripheral anterior synechia, and secondary glaucoma by positioning the IOL further away from anterior segment structures [7–9]. Currently, intrascleral fixation of posterior chamber IOL implantation is widely performed because of its safety, efficacy, and stability.

The intrascleral IOL fixation technique can be broadly classified as either sutured or sutureless, depending on the technique used to affix the haptics to the sclera [10]. In particular with suture-fixed intrascleral IOL, inflammation, suture degradation or delayed IOL dislocation by suture breakage or exposure have been reported [8]. Sutureless techniques might result in haptic damage or dislocation from the intrascleral tunnel, but do not have suture-related complications. These techniques are less time consuming, easier to perform, and retain the advantages of intrascleral IOL fixation [11, 12]. Sutureless intrascleral IOL fixation technique was first formulated by Gabor and Pavlidis in 2007 and further detailed by Agarwal and colleagues. Additionally, several modifications to this technique have been reported [13–15]. Among those techniques, Yamane developed a double-needle technique and a flanged IOL fixation technique; these two techniques can provide good IOL fixation with firm haptic fixation without using special surgical instruments and sutures. Meanwhile, these two techniques have the advantages of being simple and requiring less surgical time [1, 2]. However, several remaining issues need to be resolved. First, the IOL haptic externalization procedure might be too complicated to perform and could cause deformation of the IOL haptics, especially in eyes with a small pupil [2, 3, 16, 17]. Second, previously reported methods require many manipulations in the anterior chamber, potentially causing anterior segment complications, such as corneal decompensation [18]. Third, there are potential risks of the IOL falling into the vitreous cavity, haptic extrusion or exposure to the external environment, which might lead to endophthalmitis. Therefore, performing intrascleral IOL fixation, a relatively sophisticated technique, might be difficult for beginners or surgeons with limited experience. A longer learning curve might be required [19].

Here, we report a reliable surgical procedure, a 27-gauge needle-guided intrascleral IOL fixation with built-in 8–0 absorbable sutures. This technique requires no special instruments for IOL fixation and fewer anterior chamber manipulations. Moreover, the haptics of the IOL can be easily externalized through sclerotomy. We performed this technique in a series of eyes without sufficient posterior capsular support, such as those with complex ocular trauma or other aphakia. Based on our results, this technique provides good IOL fixation with acceptable wound closure.

## Methods

Fourteen eyes of 14 patients who underwent 27-gauge needle-guided intrascleral IOL fixation with built-in 8–0 absorbable sutures between May 2017 and July 2018 were retrospectively evaluated. The inclusion criteria were secondary implantation of intrascleral-fixated IOL for aphakia, dislocated IOL, subluxated or dislocated crystalline lens without sufficient capsular support; patients with previous additional surgical procedures were also included, such as primary pars plana vitrectomy (PPV), lensectomy plus silicone oil tamponade for ocular trauma, retinal detachment (RD), or proliferative diabetic retinopathy (PDR). Additional criteria included an improved best-corrected visual acuity (BCVA) after previous surgical operations, preoperative corneal endothelial cell density (ECD) of at least 800 cells/mm^2^, and agreement with the study protocol. The exclusion criteria were preoperative intraocular pressure (IOP) of 25 mmHg or more while receiving antiglaucomatous agent treatment, scleritis, and a postoperative follow-up duration of less than 6 months [7, 12]. All surgeries were performed by the same experienced surgeon (Z.Y. W.) at Shanghai Ninth People’s Hospital, Shanghai Jiao Tong University School of Medicine. This study adhered to the tenets of the Declaration of Helsinki. Ethics Committee approval was obtained from the Shanghai Ninth People’s Hospital review board. Informed consent was obtained from all patients or their parents, and the possible complications of the procedure were explained.

We obtained medical records containing information on demographics and the reason for surgery. Standard ophthalmologic examination records, such as the initial BCVA, slit-lamp evaluation (SL-D7, Topcon, Tokyo, Japan), IOP, follow-up duration, final visual acuity, corneal ECD, postoperative IOL position, spectral domain optical coherence tomography (OCT) (TR-KT-2913, Heidelberg Engineering GmbH, Heidelberg, Germany) and ultrasound biomicroscopy (UBM) (SW-3200, Suoer, Tianjin, China) were also obtained.

Accurate positioning of the IOL was measured by IOL tilt and decentration. A straight line between the iris-corneal angles was marked as the reference line. IOL tilt was defined as the angle between the reference line and the horizontal axis of the IOL. IOL decentration was defined as the horizontal distance between the midpoint of the reference line and IOL horizontal axis [2, 7]. Both the horizontal and vertical UBM images were used to analyze the mean IOL tilt and decentration.

The Wilcoxon signed-rank test was used to determine the significance of any association between preoperative and postoperative BCVA or ECD. A *p*-value less than 0.05 was considered significant. Statistical analyses were performed using SPSS for Mac software (version 25.0, IBM Corp., NY, USA).

### Surgical technique

Under preoperative preparation and peribulbar anesthesia, two one-half to two-thirds thickness limbal-based scleral flaps (3.0 × 3.0 mm) were created at the 4 o’clock and 10 o’clock positions. A superior 3.0 mm corneal incision was created. An infusion cannula or anterior chamber maintainer was inserted to protect the corneal endothelium.

**Fig. 1:**
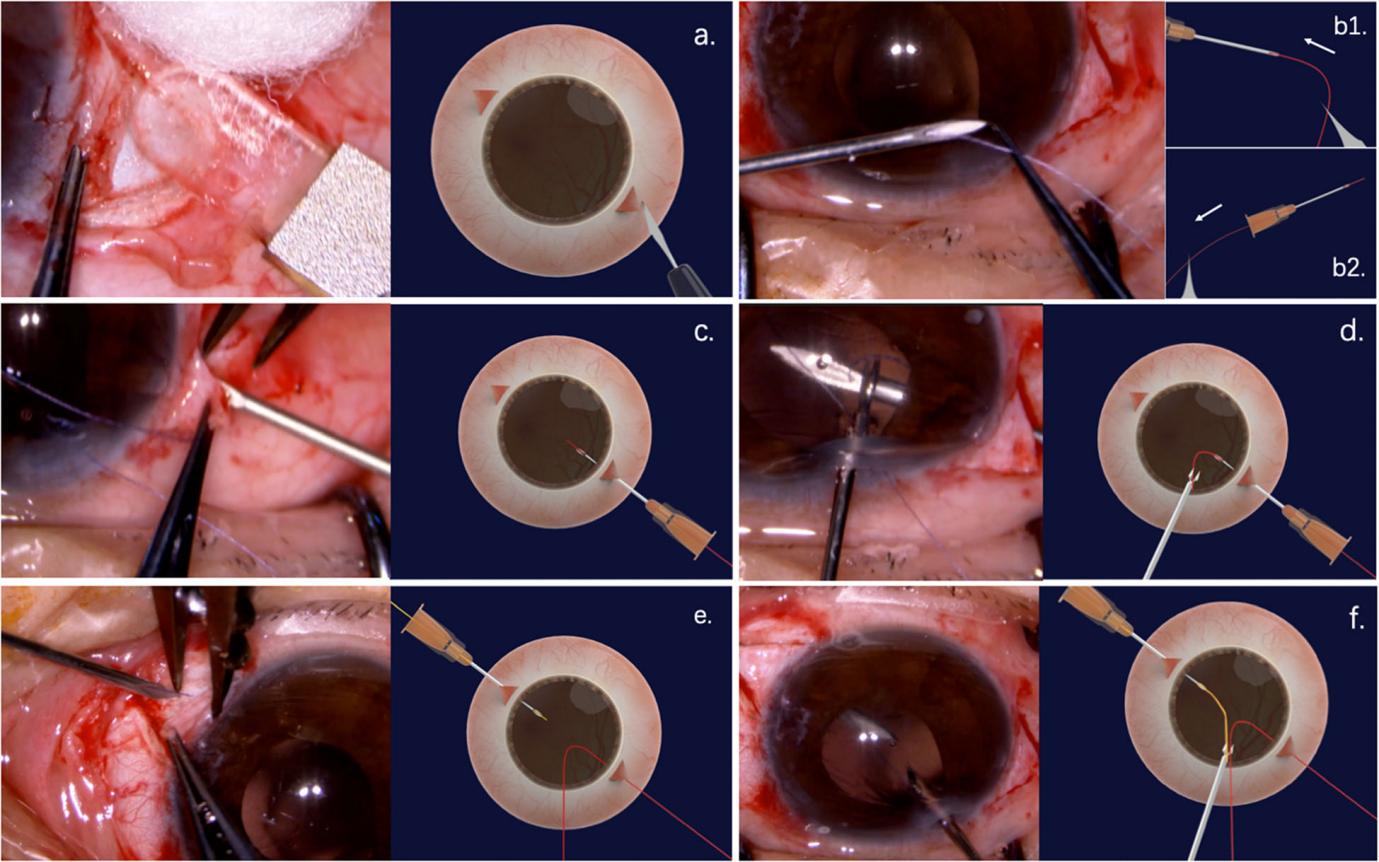
The 27-gauge needle-guided intrascleral IOL fixation technique with 8–0 absorbable sutures. **a**. Two one-half to two-thirds thickness limbal-based scleral flaps were created. **b1**. Absorbable suture was inserted into a 27-gauge round needle. **b2**. Almost all of the absorbable suture was inserted into a 27-gauge needle with only a small portion out of the needle for easy traction. **c**. The needle was used to create a sclerotomy at the 10 o’clock position under the previously created scleral flap. **d**. Forcep was used to grasp the 8–0 absorbable suture and externalize the suture through the previously created 3.0 mm corneal incision. **e**.**f**. 4 o’clock manipulation

The 8–0 absorbable sutures (L-2748 K, Covidien, Massachusetts, USA) (Additional file 1) were inserted into 27-gauge round needles, which were used to create sclerotomies at 2.0 mm from the limbus, 180 degrees apart diagonally, under the previously created scleral flaps. Forceps were used to grasp the 8–0 absorbable sutures and externalize the sutures through the previously created 3.0 mm corneal incision. A 3-piece IOL (AR40e, Advanced Medical Optics, Santa Ana, America) was placed into the injector, the end of the leading haptic was extruded and cauterized to create a small flange using an ophthalmic cautery device. (Fig. 1) The 4 o’clock 8–0 suture was then used to tie several knots at the end of the leading haptics. Satisfactory suture fixation could be achieved by making the first flanged end approximately 1.2 times larger than the bare haptics. The main purpose of this flanged end was to allow the 8–0 suture to anchor. Then, the 3-piece IOL was inserted into the anterior chamber by the injector, and the trailing haptic was kept outside to prevent the IOL from falling into the vitreous cavity. The trailing haptic was cauterized (1.2 times larger), tied to the 10 o’clock 8–0 suture and then carefully inserted into the anterior chamber. A U-hook was used to guide the IOL to the center of the pupil. The sutures were then grasped with forceps to guide haptics externalization through the sclerotomies.

**Fig. 2:**
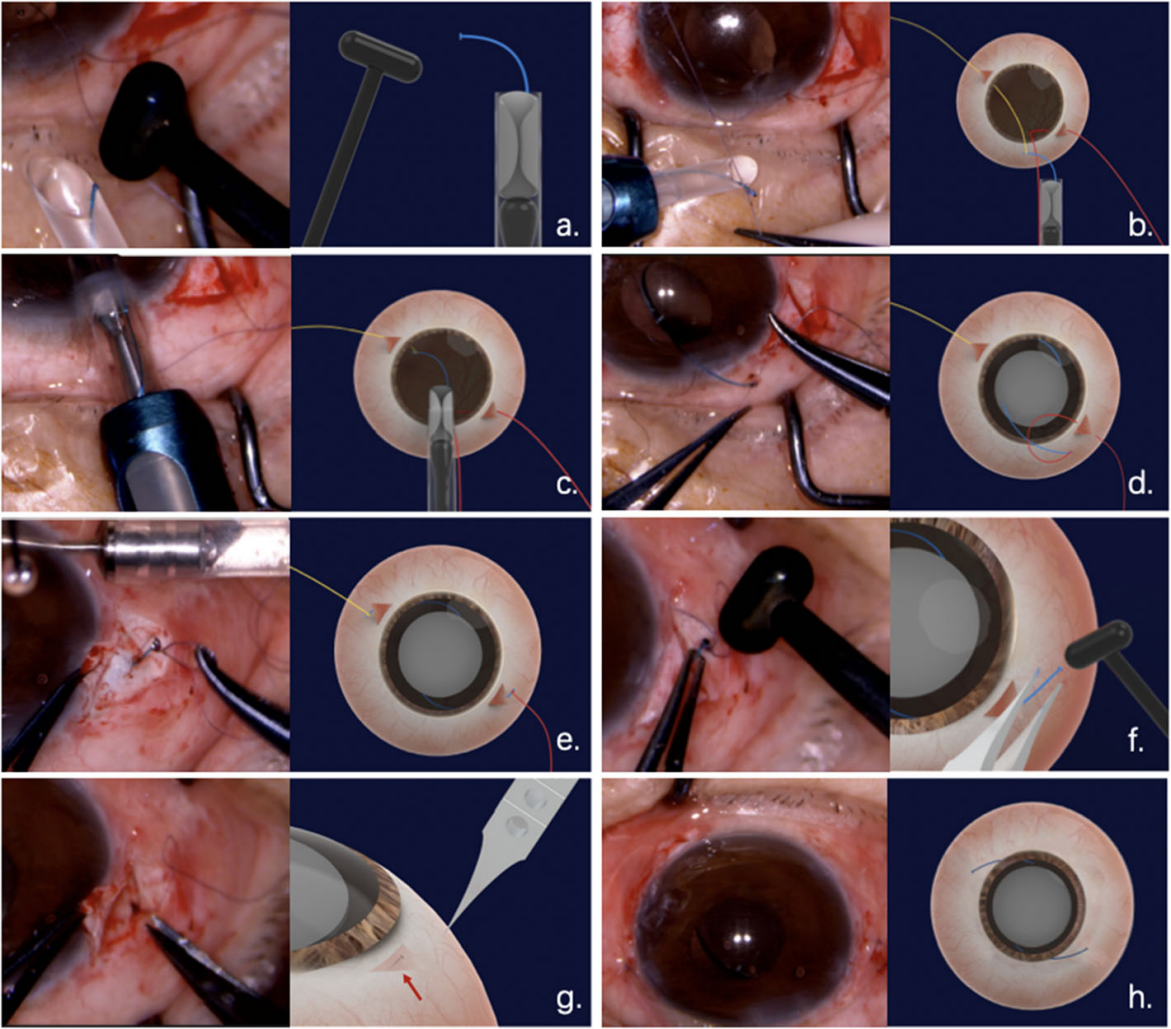
Surgical procedure of 27-gauge needle-guided intrascleral IOL fixation technique with 8–0 absorbable sutures. **a** A 3-piece IOL was placed into the injector, the end of the leading haptic was extruded and cauterized to create a small flange (1.2 time larger) using an ophthalmic cautery device. **b** Several knots were tied at the end of the leading haptic. **c** The IOL was inserted into the anterior chamber, and the trailing haptic was kept outside to prevent the IOL from falling into the vitreous cavity. **d** The trailing haptic was cauterized (1.2 times larger), tied to the 10 o’clock 8–0 suture and then carefully inserted into the anterior chamber. **e** The sutures were then grasped with forceps to guide haptics externalization through the sclerotomies. **f** A sufficient flange (1.5 times larger) was created at the end of the haptics to prevent the haptics from slipping off. **g** Each flange of the haptics was pushed back and fixed into previous needle-created scleral tunnel. **h** Closing of the scleral flaps



After externalization, a sufficiently larger flange was created at the end of the haptics and inserted into the sclera tunnel for firm fixation. According to our experience, satisfactory IOL fixation can be achieved by making the second flanged end approximately 1.5 times larger than the bare haptics. The main purpose of this flange is to prevent the haptics from slipping off. The haptics were then buried under 3.0 mm scleral flaps. Then the scleral flaps were closed by sutures or fibrin glue [15] (Fig. 2). Topical steroids were used in all patients postoperatively. The duration of surgery was recorded by video. (Additional file 2: Video S1).

**Additional file 2: Video S1.**



## Results

The haptics were well fixed and the IOL was centrally positioned in all 14 eyes of 14 patients (11 males, 3 females; mean age, 45.86 ± 19.14 years old; range, 7–74 years old). The mean follow-up period was 9.57 ± 2.87 months (range, 6–15 months). All patients had associated ocular conditions such as complicated ocular trauma (9 eyes), PDR (3 eyes) and primary RD (2 eyes). Some patients had complications before surgery (e.g., iridodialysis, traumatic glaucoma or endophthalmitis).

The BCVA was measured as the total number of letters on the Early Treatment Diabetic Retinopathy Study (ETDRS) visual acuity chart when assessed at a starting distance of 4 m. When comparing the last follow-up to preoperative visual acuity, the mean change in the BCVA was + 26.32 letters. The mean BCVA was 1.18 ± 0.70 logarithm of the minimum angle of resolution (logMAR) units preoperatively and 0.64 ± 0.60 logMAR units at the last follow-up visit postoperatively, which were statistically significantly differences (*P* = 0.011). However, the BCVA did not improve in 4 cases during the follow-up period. In 2 cases, the patient had severe PDR with poor visual acuity before surgery. In 1 case, the patient had complicated ocular trauma. In 1 case, the patient had rhegmatogenous retinal detachment (RRD). Since nearly two-thirds of the patient had ocular trauma and the other patients had either RD or PDR, some of our patients had unsatisfactory visual outcomes because of their underlying pathology.

The postoperative corneal ECD decreased from 2183 cells/mm^2^ to 2024 cells/mm^2^ (*P* < 0.01), and the rate of mean endothelial cell loss was 7% ± 6% at the last follow-up visit. The horizontal tilt and decentration at the last follow-up visit were 2.23 ± 1.06^°^ and 298 ± 122 μm, respectively, while the vertical tilt and decentration were 2.31 ± 1.22^°^ and 292 ± 133 μm, respectively. The mean IOL tilt and decentration were 2.27 ± 1.12^°^ and 295 ± 125 μm, respectively.

Postoperative complications included postoperative hypotony in 3 eyes and transient ocular hypertension in 2 eyes, which resolved within 1 month without further significant complications. No other major perioperative or postoperative complications (e.g., wound leakage, vitreous hemorrhage, endophthalmitis, CME, serous choroidal detachment, or RD) were detected during the follow-up period. Table 1 shows preoperative and postoperative medical records of the patients.

**Table 1:** Preoperative and postoperative medical records of the patients

Cases	Sex	Age- Ranges	Eye	Preexsisting ocular disease	Preoperative BCVA	Postoperative BCVA	Follow-up (Months)	Postoperative complications
1	M	30–35	R	aphakia, OT, globe rupture, iridodialysis	0.15	0.00	15	–
2	M	45–50	L	CLS, OT, RD	0.22	0.10	8	–
3	M	20–25	R	CLS, OT, glaucoma, MH	2.00	0.40	13	transient ocular hypertension(25 mmHg)
4	M	45–50	R	CLS, OT, iridodialysis	1.30	0.10	9	transient ocular hypertension(26 mmHg)
5	F	55–60	L	CCE, PDR,VH, RD_*_	1.30	1.52	8	–
6	M	30–35	L	CLD, OT, VH, RD, iridodialysis	2.00	1.52	14	–
7	F	60–65	L	aphakia, PDR, VH	2.00	0.40	11	postoperative hypotony(5 mmHg)
8	M	60–65	L	aphakia, RRD_*_	0.52	0.52	10	–
9	M	25–30	R	aphakia, OT, iridodialysis, sympathetic ophthalmia_*_	0.40	0.52	6	postoperative hypotony(3 mmHg)
10	F	55–60	R	aphakia, RRD, high myopia	1.52	0.70	8	postoperative hypotony(7 mmHg)
11	M	65–70	L	CLS, OT, retinal tear	1.52	0.40	8	–
12	M	70–75	L	aphakia, PDR_*_	2.00	2.00	7	–
13	M	5–10	L	aphakia, OT, endophthalmitis	0.70	0.52	11	–
14	M	45–50	L	CCE, OT	0.82	0.30	6	–

*M* male, *F* female, *OT* ocular trauma, *CLS* crystalline lens subluxation, *CCE* concomitant cataract extraction, *CLD* crystalline lens dislocation, *RRD* rhegmatogenous retinal detachment, *RD* retinal detachment, *MH* macular hole, *PDR* proliferative diabetic retinopathy, *VH* vitreous hemorrhage, *BCVA* best-corrected visual acuity

_*_ BCVA did not improve

## Discussion

The ideal placement of an IOL is within the capsular bag, the anatomical position [20]. However, in eyes with inadequate capsular support, the intrascleral posterior chamber IOL fixation technique is advantageous over other IOL implantation techniques because of its stability and proximity to the physiological anatomical position of the original lens [1–3, 10]. The most common indications for this procedure include posttraumatic aphakia, aphakia after complex cataract surgery, or lensectomy during complex surgical procedures, such as RD repair, PDR, IOL dislocation, or crystalline lens subluxation [21].

The various intrascleral IOL fixation techniques can be broadly classified as either sutured or sutureless. Sutureless techniques for scleral IOL fixation have advantages because they do not lead to suture degradation, late IOL dislocation caused by broken sutures or other suture-related complications [11]. The critical difference between these techniques is the manner in which the haptics of the IOL are handled [22]. Gabor and Agarwal et al. achieved sutureless scleral IOL fixation using fibrin glue to close the scleral flaps [14, 15]. Ohta et al. created a Y-shaped scleral incision to fix the haptic without using fibrin sealant [3, 18]. Yamane et al. developed a double-needle technique and flanged IOL fixation technique to provide firm haptic fixation without using suture or glue [1, 2].

There are two surgically challenging steps in intrascleral-fixated IOL procedures [13]. The first is externalization of IOL haptics. The intraocular forceps technique was reported by Gabor and Pavlidis, [14] but this technique might cause deformation of the IOL haptics. The double-needle technique, which was reported by Yamane, might make it difficult to grasp the second haptic and insert it into a scleral tunnel after the first haptic is externalized [1, 2]. Therefore, compared with other techniques, our approach solves the problem of the difficulty in grasping the second haptic after externalization of the first haptic. Meanwhile, it can minimize the risk of multiple anterior segment manipulations. It is a simple and reliable surgical technique that is suitable for beginners and surgeons with limited experience. Each step of this technique is simple, and it is easy to perform with a short learning curve. In our procedure, a disposable 27-gauge syringe needle is used to create the sclerotomy, and a lead 8–0 suture is inserted into the posterior chamber to guide haptic externalization. During externalization, there is no risk of the IOL falling into the vitreous cavity because the sutures were used to tie knots at the end of each haptics. Since large diameters sclerotomy can result in wound leakage and postoperative hypotony, we used 27-gauge needles, which caused minimal damage to the conjunctiva and sclera and created a self-sealing sclerotomy wound [23]. No other intraocular surgical instruments or manipulations were required at this step, which minimized possible damage to the cornea, peripheral retina, and other intraocular tissues. Fewer anterior segment manipulations may result in faster postoperative visual rehabilitation and a lower risk of anterior segment complications, such as corneal decompensation [24, 25].

The second surgically challenging step in this procedure is fixation of each IOL haptic inside the scleral tunnel. Intrascleral IOL fixation techniques could also be classified into those with and without a scleral flap [2, 3]. Techniques without a scleral flap are simpler and do not require sutures or fibrin glue. However, there is a potential risk of haptic extrusion. Unstable intrascleral fixation may cause IOL decentration or dislocation, which may impact refraction and visual function [26, 27]. IOL haptic fixation is easily accomplished using techniques with a scleral flap, but the surgical procedure is relatively complex [2]. However, the possibility of the haptics extrusion and the IOL slipping into the vitreous cavity by simply using flanged end fixation can be well prevented by the scleral flap [19, 28]. Making appropriately flanged ends is a critical process in our approach. During our procedure, we flanged the haptic ends prior to placement into the anterior chamber. The main purpose of the first flange is to allow the 8–0 sutures to anchor. We learned that satisfactory suture fixation can be achieved by making the first flanged end approximately 1.2 times larger than the bare haptics and that externalization of the haptics is less affected by flanged ends of this size. The diameter of the haptics of the 3-piece IOL is 0.14 to 0.17 mm, the outer diameter of the 27 gauge needle is 0.42 mm, and the diameter of the 8–0 absorbable sutures is less than 0.01 mm [1, 3, 29]. It is larger enough for the needle-created incision to retrieve the haptics after the 1.2 times larger flange creation and “several knots” of the sutures tied. There is resistance when the flanged end passes through the sclerotomy, especially at the moment of scleral breakthrough. As a result, we made scleral flaps, about one-half to two-thirds thickness, to decrease friction. Therefore, the tension of the sutures is sufficient to guide the IOL haptics through the scleral tunnel without the suture breaking or the knot slipping. In the final stage of the surgery, a sufficient flange, approximately 1.5 times larger than the bare haptics, was created at the end of each haptic using an ophthalmic cautery device. Then, each flange of the haptics was pushed back and fixed into previous needle-created scleral tunnel. The size of the flange is sufficient to prevent the haptic slipping through the tunnel because of the elasticity of the scleral tissue [1]. Moreover, the haptics were buried under 3.0 mm scleral flaps, which minimizes the risk of haptic extrusion or dislocation, and prevents the IOL from falling into the vitreous cavity. This technique achieved a secure and stable fixation of the haptic in the intrascleral tunnel.

**Fig. 3:**
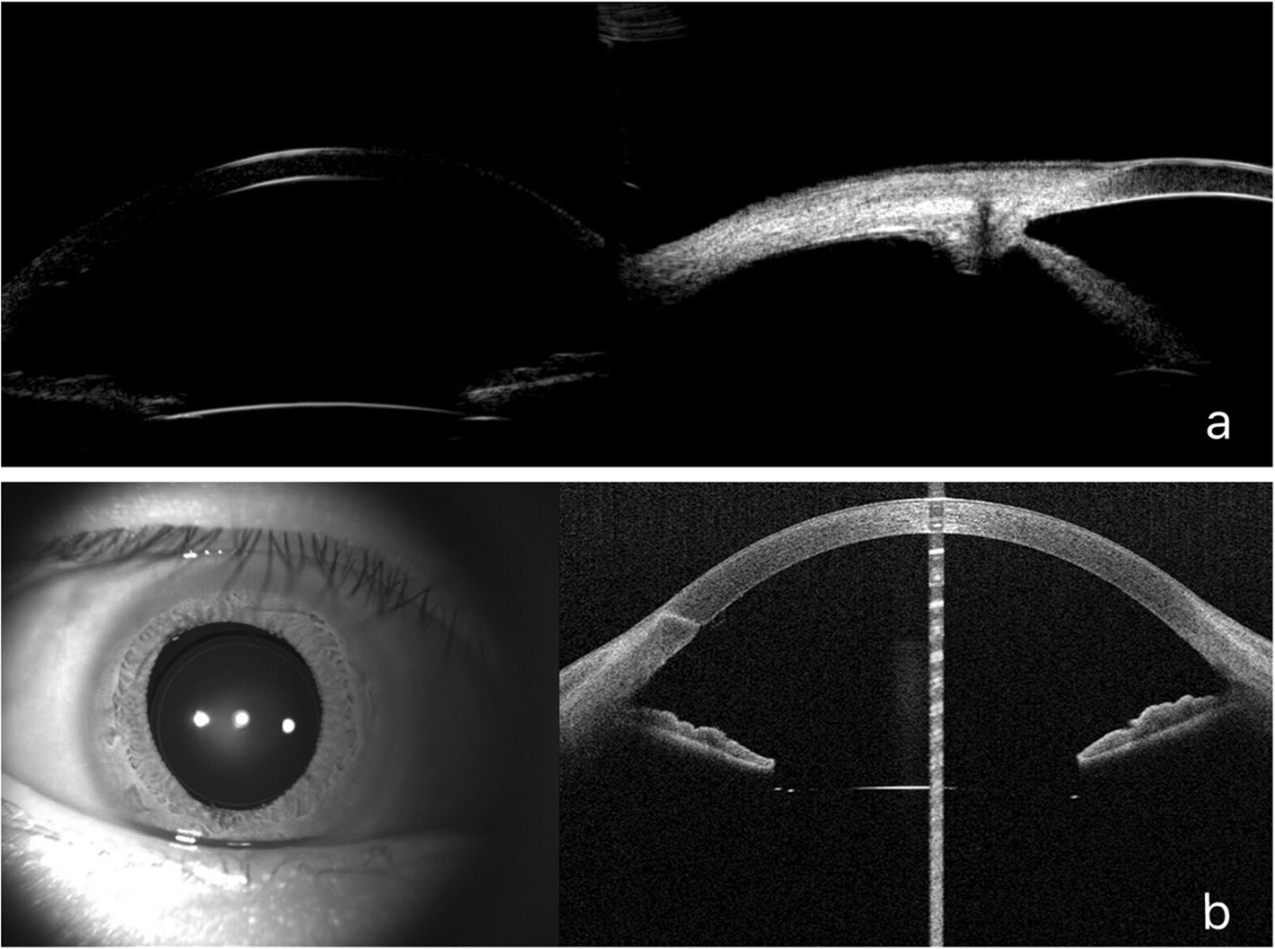
Clinical records of a representative patient at 13 months after surgery (case 3) **a** UBM image. **b** Anterior segment OCT image

According to Sindal, eyes with posttraumatic aphakia have better visual outcomes after scleral-fixated IOL implantation. Although there can be long-term suture-related complications from IOL implantation, including suture degradation or breakage associated with IOL decentration or dislocation, no differences in the outcomes or complication rates were observed between sutured and sutureless sclera-fixated IOL implantation techniques [12]. Considering the scleral flaps that we made previously, we believed that closure with sutures was needed in our procedure. The main purpose of the suturing is to close the scleral flaps, which can be replaced by the use of fibrin glue [15]. However, there is little possibility of suture erosion-related IOL decentration because the haptics of IOL were fixated by flanged end rather than sutures. UBM and anterior segment OCT demonstrated a securely fixated IOL and well-centered optic (Fig. 3).

Both primary and secondary intrascleral-fixated IOL implantation are associated with favorable visual outcomes [30]. However, we prefer to perform IOL placement after the primary surgery to address the coexistent clinical condition. Lee et al. found that eyes undergoing primary IOL implantation may have a greater risk of postoperative inflammation with associated complications like CME [12, 30]. Compared with primary scleral-fixated IOL placement, secondary IOL implantation seems to have a lower early complication rate in complicated cataract extraction, although the final visual acuity and late complication rate are not significantly different [30].

All 14 patients underwent primary vitrectomy surgery, lensectomy with or without silicone oil tamponade for the treatment of a coexisting clinical condition (e.g. ocular trauma, RD, PDR, or lens dislocation). Primary three-port PPV was performed because all of our patients required complicated retinal surgery. When complicated with cataracts, these ocular diseases are the major causes of severe visual impairment. For eyes lacking sufficient capsular support, we recommended anterior vitrectomy or PPV before IOL fixation. Vitreous incarceration and traction caused by the sutures or the IOL haptics can be prevented by vitrectomy surgery [1]. Meanwhile, a complete vitrectomy with shaving of the vitreous base can release vitreoretinal traction to avoid postoperative retinal tear or detachment [12, 31].

Although our patients had coexistent ocular conditions, the postoperative BCVA was significantly different from the preoperative BCVA (*P* = 0.011). However, the BCVA did not improve in 4 cases during the follow-up period. Two patients had severe PDR with poor glycemic control, one patient had long-standing RD before the primary operation, and the other patient suffered from a complicated ocular trauma. We believe these patients had severe visual impairment and unsatisfactory visual outcomes because of their complicated ocular and systemic diseases.

Tilt and decentration are also important predictors of accurate IOL positioning [7]. According to Holladay, spherical aberration resulting from abnormal IOL positioning was sufficient to decrease the visual acuity when the tilt was more than 7° and the decentration was more than 400 μm [7, 32]. The mean tilt (2.27 ± 1.12^°^) and decentration (295 ± 125 μm) in our study are similar to those in other studies, suggesting that the impact on the optic system is acceptable [1, 2, 6, 7, 33]. The rate of mean endothelial cell loss was 7% ± 6%. Multiple mechanisms may be involved in corneal endothelial cell loss, such as surgical injury, systemic diseases, and intraocular perfusion during surgery. Since the endothelial cell counts begins to stabilize approximately 1 year after surgery, a longer follow-up observation period is required [7].

According to a large retrospective study by Todorich, the most common complications after intrascleral-fixated IOL surgery were VH and CME [25]. The major postoperative complications of our procedure were postoperative hypotony (3 of 14 cases) and transient ocular hypertension (2 of 14 cases), which returned to the normal intraocular pressure range within one month without further complications. Postoperative hypotony has been reported as a common complication of the intrascleral IOL fixation technique. Larger corneoscleral incision and sclerotomy incisions may carry the potential risk of transient postoperative wound leakage [1, 6]. In our cases, hypotony resolved spontaneously without any intervention. Postoperative ocular hypertension could be explained by mild viscoelastic material retention and steroid response. Transiently IOP elevations were controlled by antiglaucoma medications without affecting the final visual outcome [34]. Since vitrectomy surgery has been previously performed in all cases, no cases of VH or CME were detected during the follow-up period.

There are limitations to our study, including the small sample size, limited follow-up period, and lack of a control group. A longer follow-up period is needed to further assess corneal endothelial loss, long-term IOL stability and postoperative complications. In these 14 cases, there was no evidence of IOL decentration or dislocation, no severe complications, and no cases of haptic erosion during the follow-up period.

## Conclusions

In conclusion, our 27-gauge needle-guided intrascleral IOL fixation technique with 8–0 absorbable sutures might be useful for IOL implantation in eyes without sufficient capsular support. This technique is easy to perform, achieves both anatomical and optical stability, and has fewer potential risks of IOL decentration and dislocation. However, a longer follow-up observation period is required to examine the long-term anatomical and functional outcomes associated with this technique.

## Supplementary information

**Additional file 1.** The image of the suture material: POLYSORB 8–0 (0.4 metric), 12″ (30 cm), violet braided absorbable sutures (L-2748 K, Covidien, Massachusetts, USA).

## Data Availability

The datasets used and/or analysed during the current study are available from the corresponding author on reasonable request.

## Abbreviations

ACIOL: anterior chamber intraocular lens
BCVA: best corrected visual acuity
CCE: concomitant cataract extraction
CLD: crystalline lens dislocation
CLS: crystalline lens subluxation
CME: cystoid macular edema
ECD: endothelial cell density
ETDRS: Early Treatment Diabetic Retinopathy Study
F: female
IOL: intraocular lens
IOP: intraocular pressure
logMAR: logarithm of the minimum angle of resolution
M: male
MH: macular hole
OCT: optical coherence tomography
OT: ocular trauma
PDR: proliferative diabetic retinopathy
PPV: pars plana vitrectomy
RD: retinal detachment
RRD: rhegmatogenous retinal detachment
UBM: ultrasound biomicroscopy
UGH: uveitis-glaucoma-hyphema
VH: vitreous hemorrhage
