# Supplementary figures and images for: A modified intrascleral intraocular lens fixation technique with fewer anterior segment manipulations: 27-gauge needle-guided procedure with built-in 8–0 absorbable sutures
Source: BMC Ophthalmol. 2019 Nov 21;19:234. doi: 10.1186/s12886-019-1239-2 (PMC6873762; doi:10.1186/s12886-019-1239-2)

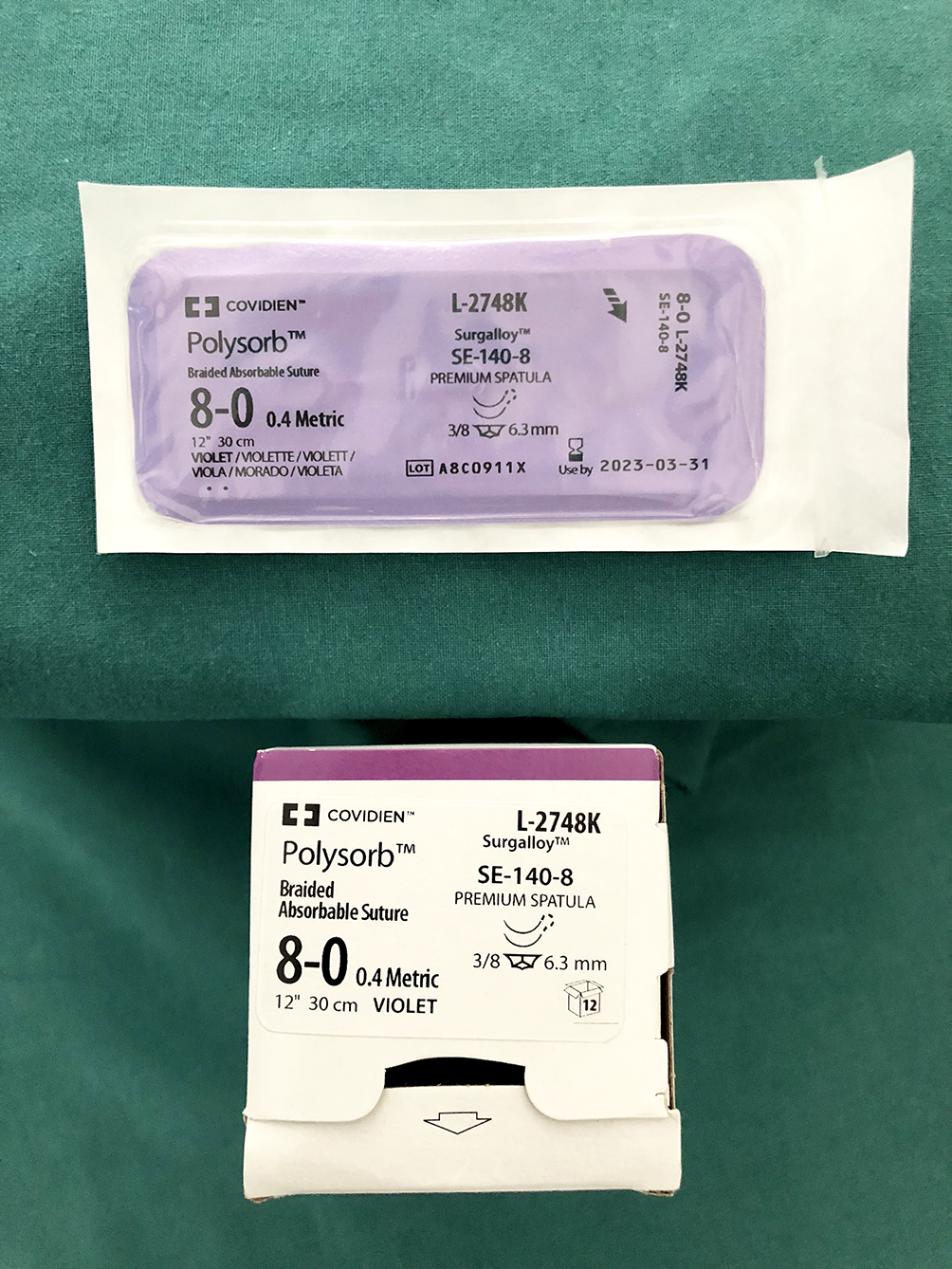

Supplement: Supplementary file 2 — Additional file 1. The image of the suture material: POLYSORB 8–0 (0.4 metric), 12″ (30 cm), violet braided absorbable sutures (L-2748 K, Covidien, Massachusetts, USA). [file 12886_2019_1239_MOESM1_ESM.tif]
